# Importance of Metabolic Adaptations in *Francisella* Pathogenesis

**DOI:** 10.3389/fcimb.2017.00096

**Published:** 2017-03-28

**Authors:** Jason Ziveri, Monique Barel, Alain Charbit

**Affiliations:** ^1^Sorbonne Paris Cité, Université Paris DescartesParis, France; ^2^Institut National de la Santé et de la Recherche Médicale U1151 - Centre National de la Recherche Scientifique UMR 8253, Institut Necker-Enfants Malades, Team 11: Pathogenesis of Systemic InfectionsParis, France

**Keywords:** *Francisella tularensis*, metabolism, glycolysis, nutrient uptake, virulence

## Abstract

*Francisella tularensis* is a highly infectious Gram-negative bacterium and the causative agent of the zoonotic disease tularemia. This bacterial pathogen can infect a broad variety of animal species and can be transmitted to humans in numerous ways with various clinical outcomes. Although, *Francisella* possesses the capacity to infect numerous mammalian cell types, the macrophage constitutes the main intracellular niche, used for *in vivo* bacterial dissemination. To survive and multiply within infected macrophages, *Francisella* must imperatively escape from the phagosomal compartment. In the cytosol, the bacterium needs to control the host innate immune response and adapt its metabolism to this nutrient-restricted niche. Our laboratory has shown that intracellular *Francisella* mainly relied on host amino acid as major gluconeogenic substrates and provided evidence that the host metabolism was also modified upon *Francisella* infection. We will review here our current understanding of how *Francisella* copes with the available nutrient sources provided by the host cell during the course of infection.

## Introduction

**Figure 1:**
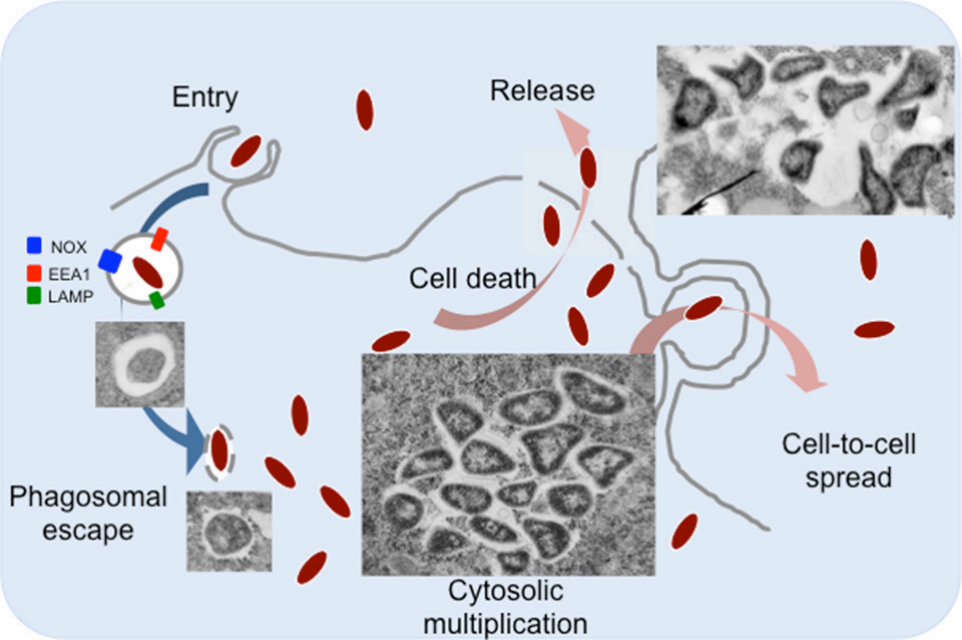
**The intracellular life cycle of ***F. tularensis*****. *Francisella* is internalized into macrophages by large pseudopodia. Inside cells, bacteria transiently reside in a phagosomal compartment that partially matures into a late phagocytic compartment, acquiring membrane markers of early (EEA1) and late endosomes/lysosomes (LAMP-1 and -2). Typical transmission electron microscopy images of intracellular *Francisella* are shown. Once in the host cell cytoplasm, *Francisella* takes advantage of available nutrients to actively multiply. Bacteria can be released from dead cells (by apoptosis and/or pyroptosis) or can be directly transferred by trogocytosis to neighboring cells.

*Francisella tularensis* is a small Gram-negative bacterium, causative agent of the zoonotic disease tularemia (Sjostedt, 2011). This facultative intracellular pathogen can infect humans by different modes, and notably direct contact with sick animals, inhalation, insect bites or ingestion of contaminated water or food (Foley and Nieto, 2010). *F. tularensis* is able to infect numerous cell types (Jones et al., 2012; Celli and Zahrt, 2013), including dendritic cells, neutrophils, macrophages as well as hepatocytes or endothelial cells but is thought to replicate *in vivo* mainly in macrophages (Santic et al., 2006). Four major subspecies of *F. tularensis* are currently listed: *tularensis, holarctica, mediasiatica, and novicida* (McLendon et al., 2006). These subspecies differ in virulence and geographical origin but all cause a fulminant disease in mice that is similar to tularemia in humans (Kingry and Petersen, 2014). Although, the subspecies *novicida* (here designated *F. novicida*) is rarely pathogenic in humans, its genome shares a high degree of nucleotide sequence conservation with the human pathogenic species and is thus widely used as a model to study highly virulent subspecies. The pathogenicity of *Francisella* is tightly associated to its capacity to multiply in the cytosolic compartment of infected macrophages (Celli and Zahrt, 2013). Different macrophage receptors involved in *Francisella* uptake have been identified (Moreau and Mann, 2013). After engulfment by phagocytic cells, *Francisella* transiently resides in a phagosomal compartment (Figure 1) that sequentially displays membrane markers of early (EEA1) and late endosomes/lysosomes (LAMP-1 and -2) but does not acquire the hydrolase cathepsin D or lysosomal tracers (Celli and Zahrt, 2013). Within the phagosome, *Francisella* must fight against several host antimicrobial defenses, including notably reactive oxygen species (ROS) produced by the NADPH oxidase (Kinkead and Allen, 2016). For this, *Francisella* is equipped with a series of enzymes that include superoxide dismutase, catalase and acid phosphatases (Jones et al., 2012). Phagosomal escape involves a number of additional factors among which its Type 6 secretion systems (T6SS) (Clemens et al., 2015; Rigard et al., 2016). The precise molecular contribution of the *Francisella* T6SS apparatus and/or effectors to phagosomal membrane disruption, as well as of additional non FPI-encoded proteins (Eshraghi et al., 2016) is not yet fully understood. The capacity of *Francisella* adaptation to the host cytosol nutritional environment has been coined “nutritional virulence” (Santic and Abu Kwaik, 2013).

*Francisella* belongs to a restricted family of bacteria that exclusively multiply in the cytosolic compartment of infected cells. This family, that notably includes *Listeria, Shigella*, and *Rickettsia*, have a direct access to the cytosolic elements necessary for their growth. In contrast, the majority of other (facultative or obligate) intracellular pathogens reside in vacuolar compartments (Creasey and Isberg, 2014) and must therefore first import their host-derived nutrients within this compartment before being able to utilize them. For example, *Legionella* takes advantage of proteasomal degradation, a natural host degradative pathway (Price et al., 2011), to obtain an abundant source of amino acids to fill-up the vacuolar compartment where it resides. Indeed, *Legionella pneumophila* has been shown to inject the effector AnkB into the infected host cells (Al-Quadan et al., 2012) which, after lipidation by the host farnesylation machinery, becomes anchored to the vacuolar membrane and serves as a platform for the assembly of Lys48-linked polyubiquitinated proteins. Proteasomal degradation then generates elevated levels of amino acids at the vacuolar membrane, which can be imported into the vacuole.

The host cytosol was initially considered as a nutrient-replete cellular compartment (Ray et al., 2009). However, numerous studies have now clearly established that it contains a number of nutrients in limiting amounts (Fonseca and Swanson, 2014; Abu Kwaik and Bumann, 2015; Eisenreich and Heuner, 2016). Invading intracellular pathogens have therefore evolved various strategies to take advantage of the available nutrient-limiting resources (Abu Kwaik and Bumann, 2013; Zhang and Rubin, 2013; Gouzy et al., 2014b,c; Miller and Celli, 2016).

After several rounds of active multiplication in the host cytosol, *Francisella* dissemination to adjacent cells occurs mainly after their release from lysed cells (Jones et al., 2012). Host guanylate-binding proteins (GBPs) have been shown to be involved in pyroptotic cell death by lysing cytosolic *Francisella* thereby, leading to the activation of the Absent in Melanoma 2 (AIM2) inflammasome (Meunier et al., 2015). Interestingly, a novel cell-to-cell dissemination mechanism has been also described very recently, by which *Francisella* can infect adjacent cells by trogocytosis (Steele et al., 2016). In this review, we will address the role of nutrient acquisition in *Francisella* intracellular adaptation and multiplication, focusing on amino acids and carbohydrates as nutrient supplies.

## Amino acids constitute a major carbon source for intracellular *Francisella*

Genome-scale genetic screens, performed in different cellular and/or animal models, have repeatedly identified genes encoding either metabolic pathways or predicted membrane proteins, highlighting the importance of metabolic adaptation and nutrient acquisition in intracellular survival of *Francisella* (Pechous et al., 2009; Meibom and Charbit, 2010). In 2009, we showed that *Francisella* used glutathione (a cysteine-containing tripeptide) as a source of intracellular cysteine to compensate its natural auxotrophy for cysteine (Alkhuder et al., 2009), thus providing the first demonstration that this pathogenic bacterium relied on host-derived compounds for intracellular survival. Because of its multiple auxotrophies (arginine, histidine, lysine, tyrosine, methionine, and cysteine), resulting from genetic alterations impairing biosynthetic pathways (Larsson et al., 2005), *Francisella* must acquire many other amino acids, including some available only in limiting concentrations within infected host cells. For this, the bacterium possesses high affinity dedicated uptake systems. Our previous genome analyses revealed that *F. tularensis* is mainly equipped with secondary carriers (Meibom and Charbit, 2010). This family of transporters encompasses several major families, including amino acid transporters, such as the amino acid-polyamine-organocation transporters (APC), the proton-dependent oligopeptide transporters (POT); the hydroxy/aromatic amino acid permeases (HAAAP); as well as the major facilitator superfamily (MFS) proteins, which is involved in a variety of transport functions, including amino acid uptake.

Since 2014, our laboratory has been able to characterize four amino acid transporters (two MFS members, AnsP and IleP; and two APC members, GadC, and ArgP) and to elucidate their contribution to the major steps of *Francisella* intracellular life cycle. We will first briefly recall below their respective properties and then discuss how amino acid availability could be used by the bacterium to sense its intracellular environment.

*GadC, AnsP, IleP, and ArgP: all for one…*We first functionally characterized the glutamate permease GadC (Ramond et al., 2014) and showed that intracellular multiplication of a Δ*gadC* mutant was essentially abolished because the mutant bacteria remained trapped within the phagosomal compartment. Specifically, our experimental data revealed that GadC contributed, within this compartment, to resistance to reactive oxygen species (ROS). Direct quantification, of tricarboxylic acid (TCA) cycle intermediates present in the cytoplasm of the wild-type and Δ*gadC* strains, showed that inactivation of the *gadC* gene significantly affected succinate, fumarate, and oxoglutarate contents. These data supported the notion that imported glutamate is used by *Francisella* to feed the TCA cycle within the phagosomal compartment (Ramond et al., 2014). Imported glutamate can be converted into various compounds like glutamine, glutathione, GABA or oxoglutarate (OG), which is known to be a potent anti-oxidant molecule (Mailloux et al., 2009). Of note, we had previously shown that the AAA+ chaperone MoxR of *Francisella* (Dieppedale et al., 2013) interacted physically with the enzymes pyruvate dehydrogenase and oxoglutarate dehydrogenase and that this interaction was required for optimal activity of these two enzymes. Since *moxR* gene inactivation also impaired bacterial intracellular viability and stress resistance, one may assume that the activity of the TCA cycle contributes to stress defense and bacterial virulence. The role of the TCA cycle in stress defenses is currently investigated in other pathogenic bacterial species and a direct role of the oxoglutarate dehydrogenase in resistance to nitrosative stress has been recently demonstrated in *M. tuberculosis* (Maksymiuk et al., 2015).

In a parallel study, we identified a second permease, designated AnsP, specifically involved in asparagine uptake. In sharp contrast to GadC, AnsP appeared to be exclusively required for bacterial cytosolic multiplication. Impaired intracellular growth of the *F. novicida* Δ*ansP* mutant could be fully suppressed upon supplementation with an excess of asparagine, *in vitro* as well as *in vivo* (Gesbert et al., 2014). Of note, Neyrolles and co-workers have shown that *M. tuberculosis*, which is prototrophic for all 20 amino acids in broth, also relied on two paralogous amino acid transporters, one for aspartate and one for asparagine (designated AnsP1 and AnsP2, respectively) for intracellular survival and multiplication (Gouzy et al., 2013, 2014a,b,c). Interestingly, genetic inactivation of the gene encoding AnsP1 led to a severe decrease of mycobacterial fitness *in vivo*. In contrast, inactivation of the asparagine transporter AnsP2, although specifically involved in asparagine uptake at acidic pH, did not affect bacterial virulence *in vivo*.

The fact that *Francisella* is prototrophic for both glutamate and asparagine during growth in synthetic medium (and possesses intact biosynthetic pathways) suggests that the bacterium becomes “phenotypically” auxotrophic in infected cells, in agreement with the notion that uptake is generally preferred to synthesis. Whereas AnsP-dependent asparagine uptake was more specifically dedicated to protein synthesis, GadC-dependent glutamate transport was required for oxidative stress defense, indicating that amino acid acquisition contributes to multiple aspects of intracellular bacterial adaptation. The mechanisms down-regulating (or not up-regulating) the corresponding biogenesis pathways remain to be elucidated in intracellular bacteria.

More recently, we showed that two other amino acid permeases, IleP, and ArgP, were required in both phagosomal and cytosolic compartments. The orthologues of IleP, in the highly virulent strain *F. tularensis* Schu S4 and in *F. tularensis* LVS, had been shown to be required for normal bacterial replication in the hepatocytic human cell line HepG2 (Qin and Mann, 2006; Marohn et al., 2012). We found that the MFS permease IleP of *Francisella* mediated isoleucine uptake and was vital for bacterial intracellular multiplication and virulence (Gesbert et al., 2015). Remarkably, inactivation of the *ileP* gene in both *F. novicida* and *F. tularensis* LVS led to a delayed bacterial phagosomal escape and to a reduced cytosolic multiplication.

Genome comparisons showed that pathogenic *Francisella* subspecies possessed defective branched-chain amino acid (BCAA) pathways involved in leucine, isoleucine, and valine biosynthesis, relying thus exclusively on transporter-mediated acquisition of these amino acids from the host. At the opposite, *F. novicida* was equipped with an intact BCAA pathway and could use threonine as a precursor for their synthesis.

Of note, AnsP, and IleP proteins both belong to the Phagosomal transporter (Pht) subclass of MFS that is exclusively found among intracellular pathogenic bacteria (Chen et al., 2008). In *L. pneumophila*, the Pht protein PhtA has been shown to be required for threonine uptake in the Legionella-containing vacuole (Sauer et al., 2005). The *L. pneumophila* genome encodes 10 additional PhtA paralogues, some of which are also required during intracellular replication (Fonseca and Swanson, 2014). Interestingly, whereas PhtJ is required for acquisition of valine (Sauer et al., 2005), PhtC and PhtD were more recently shown to contribute to protecting *L. pneumophila* from dTMP starvation (Fonseca et al., 2014), indicating that Pht transporters are not strictly devoted to amino acid uptake.

We also showed that the permease ArgP of *Francisella* was a high affinity arginine transporter (Ramond et al., 2015). ArgP-mediated arginine uptake appeared to be crucial for efficient phagosomal escape. Arginine constitutes an essential amino acid for *Francisella* since the metabolic pathways, leading to- or coming to arginine, are predicted to be inactive, thus, highlighting the importance of essential amino acids during early stage infection. By using high-resolution mass spectrometry, we found that arginine limitation affected biogenesis of the majority of the ribosomal proteins. Indeed, in bacteria grown under arginine limiting conditions, the majority of the ribosomal proteins identified (app. 80%) were present in lower amount in the Δ*argP* mutant compared to wild-type, suggesting possible links between ribosomal proteins amounts and phagosomal escape (Ramond et al., 2015). Of note, ArgP is the closest paralogue of GadC within the APC family of transporter (Meibom and Charbit, 2010).

The contribution of the glycine cleavage system (GCS) to the pathogenesis of Francisella was addressed by Gerard Nau and co-workers (Brown et al., 2014). Genes encoding the GCS have been identified in genome-wide genetic screens developed to identify novel *Francisella* virulence genes (Meibom and Charbit, 2010). This pathway facilitates the degradation of glycine to acquire 5,10-methylene-tetrahydrofolate, a one carbon donor utilized in the production of serine, thymidine, and purines. Hence, the GCS is expected to contribute to pathogen fitness in conditions where these metabolites are limiting. In *F. tularensis* Schu S4, inactivation of the glycine cleavage system aminomethyltransferase T (GcvT) leads to serine auxotrophy, thus implying that the intact *serABC* pathway still present in the Δ*gcvT* mutant strain is either insufficiently active *in vitro* or not involved in serine biosynthesis in Francisella. The authors demonstrated that the *F. tularensis* GCS was essential for intracellular multiplication in conditions of serine limitation and contributed to *in vivo* pathogenesis. Of note, the GCS has been previously associated to persistence during chronic bacterial infection with Brucella abortus (Hong et al., 2000).

## Could amino acids serve as sensors of the intracellular milieu?

The metabolism of branched chain amino acid lies at the crossroads of several other bacterial metabolic pathways in living cells. BCAAs are essential amino acids for humans and therefore must be supplied in the diet. Yet, BCAAs are among the most abundant amino acids in proteins; maintaining their pools is, thus, a prerequisite for high level synthesis of proteins. As mentioned above, in the pathogenic subsps *holarctica* and *tularensis*, BCAA degradation pathways were predicted to be nonfunctional, suggesting that exogenously acquired BCAAs could be used mainly for protein synthesis in these species. Interestingly, we found that the *F. tularensis* LVS triggered the uptake of important amounts of BCAAs upon entry into THP-1 macrophages (Gesbert et al., 2015). Indeed, the intracellular BCAA concentration sharply increased after 1 h of infection and strongly decreased after 24 h, suggesting that these amino acids had been consumed during the course of intracellular bacterial multiplication. We found that infection with *L. monocytogenes* EGD-e strain also triggered a significant (10-fold) rise in the concentration of each of the BCAAs in these cells but their concentration varied only very moderately over the course of infection. Of note, it has been previously reported that *L. monocytogenes* infection induced the BCAA pathway in macrophages (Lobel et al., 2012), suggesting that *L. monocytogenes* encountered limited amounts of BCAAs in the host cytosol. Furthermore, the pleiotropic isoleucine-responsive regulator CodY was found to be responsible for the upregulation of *L. monocytogenes* virulence genes under limiting concentrations of BCAAs in chemically defined medium. These observations led Herskovits and co-workers to propose that the limiting intracellular concentrations of BCAAs could represent a signal for the bacteria to sense their subcellular localization. More recently, these authors demonstrated that CodY directly bound the proximal portion of the coding sequence of the master virulence activator gene, *prfA*, and that this binding resulted in up-regulation of *prfA* transcription specifically under low concentrations of BCAA (Lobel et al., 2015), linking directly metabolism and virulence in this pathogen. *Francisella* genomes do not encode any CodY orthologue. Hence, one may speculate that intracellular isoleucine concentration influences *Francisella* genes expression via another, yet unidentified, regulatory mechanism.

Macrophages are able to synthesize their own arginine and to import it via constitutive and inducible cationic amino acid transporters CAT-1 (or SLC7A1) and CAT-2 (or SLC7A2), respectively (Ramond et al., 2015). Arginine serves either to produce nitrogen reactive species (NO, via NO synthases) or convert it to ornithine and urea (via type 1 arginase). The phagosome into which *Francisella* transiently resides is a dynamic compartment whose size and internal composition may considerably vary during the limited period of time spent by the bacterium. The arginine concentration available to *Francisella* in the phagosome might also progressively decrease during its maturation. One may suggest that intracellular *Francisella* respond to variations of arginine availability in this compartment by regulating their own ribosomal protein biogenesis. Indeed, repression of ribosomal protein synthesis in response to stresses, such as nutritional limitation, has been observed in all kingdoms of life (Conrad et al., 2014).

## Linking carbohydrate metabolism and amino acid uptake

In mammalian cells, glycolysis and the oxidative branch of the pentose-phosphate pathway occur in the cytosol as the anabolic reactions (gluconeogenesis; and amino acid, nucleotide, fatty acids biosynthesis). In contrast, the TCA cycle and the electron-transfer chain, leading to oxidative phosphorylation, take place exclusively in the mitochondria and metabolites, such as pyruvate, are transferred from the cytosol to the mitochondria to feed the TCA. While several intracellular bacterial pathogens (such as enteroinvasive *Escherichia coli* and *Brucella*) mainly rely on glucose as a preferred carbon source for their intracellular metabolism (Abu Kwaik and Bumann, 2015), others simultaneously use multiple carbon sources (Abu Kwaik and Bumann, 2015). For example, *L. monocytogenes* has been shown to rely on two major carbon substrates, glycerol, and glucose-6P (Eisenreich et al., 2010; Grubmuller et al., 2014), but preferentially uses glycerol during its intracellular replication. Like *L. monocytogenes, Francisella* can use glucose as a carbon and energy sources. However, *L. monocytogenes* possesses a transporter (UhpT) specifically mediating the uptake of host glucose-6P that constitutes a key element of its cytosolic multiplication (Chico-Calero et al., 2002). *Francisella* genomes do not encode any orthologue of the UhpT sugar phosphate transporter family and *F. novicida* and *F. tularensis* LVS are even unable to ferment glucose-6P (Gesbert et al., 2014). This suggests that host-derived glucose-6P might not be utilizable as a source of carbohydrate by *Francisella* during intracellular multiplication. In contrast, the non-pathogenic related species *Francisella philomiragia*, which has an environmental habitat, possesses a *uhpT* orthologous gene (Fphi_0883, unpublished observation), suggesting that this bacterium is able to utilize this sugar during its planktonic life.

Of note, *Francisella* genomes encode a glycerol uptake transporter (GlpF) and a glycerol-3 phosphate transporter (GlpT). Hence, in the absence of glucose, *Francisella* probably mainly uses the available carbon sources (such as pyruvate, glycerol and glycerol-3P), in addition to amino acids, as intracellular carbon and nitrogen sources. The mechanisms by which glucose or glycerol are taken up by *Francisella* are still unknown. Indeed, genome analyses indicate that *Francisella* is devoid of any carbohydrate PEP-dependent phosphotransferase (PTS) system (Meibom and Charbit, 2010) or other non-PTS putative glucose permease.

**Figure 2:**
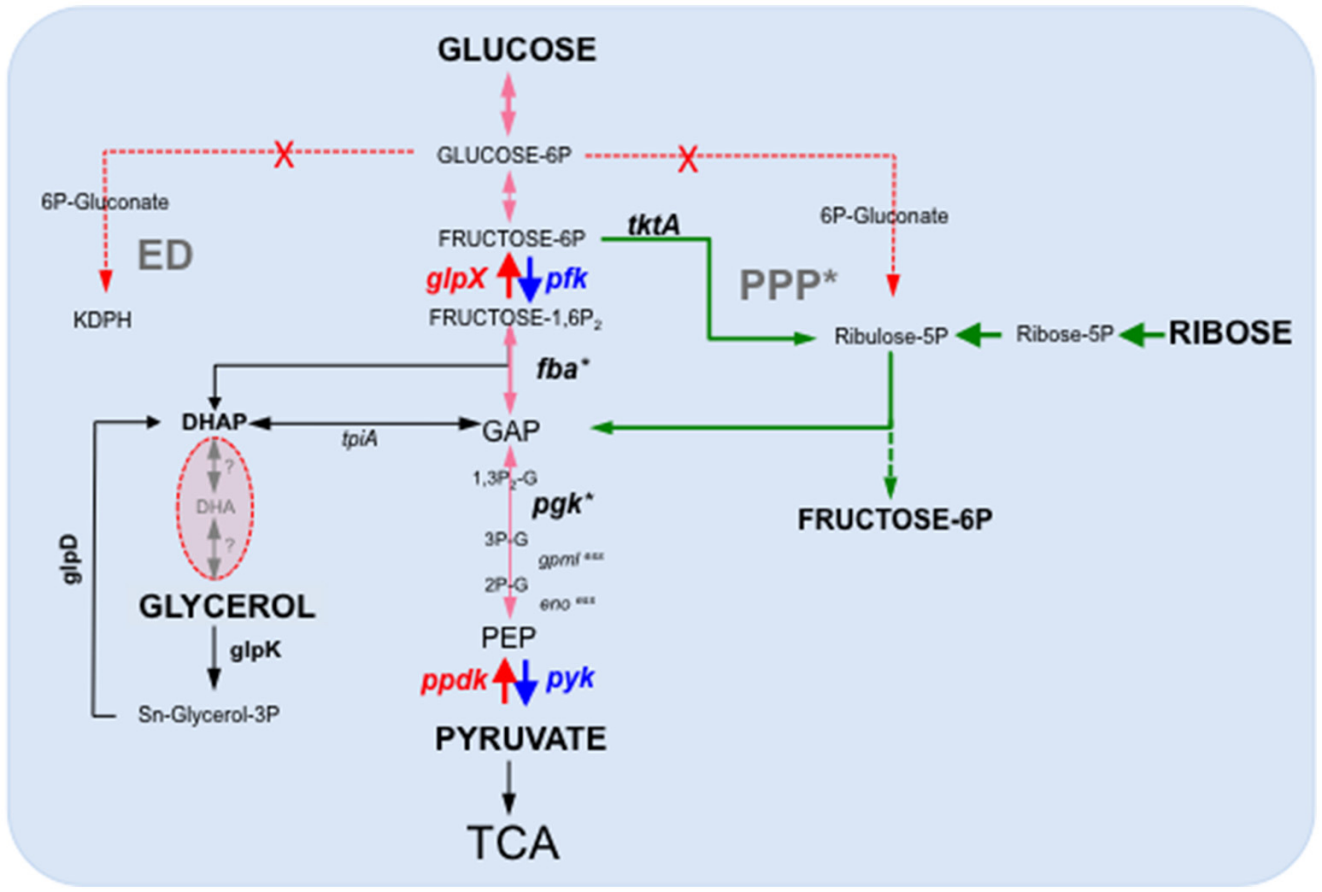
**Schematic depiction of the ***Francisella*** glycolytic and gluconeogenic pathways**. *F. tularensis* possesses a complete glycolysis and gluconeogenesis pathways as well as an intact TCA cycle. In contrast, it lacks a functional Entner-Doudoroff (ED) pathway and the oxidative branch of the Pentose phosphate pathway (PPP), in red-dotted arrows. PPP^*^, the non-oxidative branch of the PPP is still functional. Ppdk and FBPase (in red) represent only two enzymatic steps specifically acting in the gluconeogenic direction. The dotted green arrows indicate the non-oxidative part of the Pentose Phosphate Pathway. Gene numbers in *F. novicida*: *pgm* (*FTN_0514*); *pgi* (*FTN_0663*); *gpml* (*FTN_0648*); *eno* (*FTN_0621*); *glpX* (*FTN_0298*); *pfk* (*FTN_1210*); (*FTN_1631*); *glpD* (*FTN_1584*); *glpK* (*FTN_1585*). *x*^ess^, essential genes. ^*^Expression of *fba* and *pgK* have shown to be up-regulated in BMM (Wehrly et al., 2009). *F. tularensis Schu S4* and *F. novicida* U112 are able to ferment glycerol but the enzymes involved are unknown. *F. tularensis LVS* is unable to ferment glycerol. DHAP, Dihydroxyacetone phosphate (or Glycerone-Phosphate); GAP, glyceraldehyde 3-phosphate; PEP, phosphoenolpyruvate.

Gluconeogenesis, which allows glucose synthesis from non-sugar compounds such as amino acids or TCA cycle intermediates, is involved in virulence of several intracellular bacterial pathogens, including *M. tuberculosis* (Marrero et al., 2010; Puckett et al., 2014) but is dispensable for other bacteria such as *Brucella abortus* (Zuniga-Ripa et al., 2014). In *Francisella*, we recently showed that gluconeogenesis constituted a major pathway required for pathogenesis (Brissac et al., 2015). Indeed, inactivation of the gene *glpX*, encoding the unique class II fructose biphosphatase (FBPase) of *Francisella*, severely impaired intra-macrophagic bacterial multiplication, in the presence of gluconeogenic substrates and considerably attenuated virulence in the mouse mode. The strictly gluconeogenic enzyme is responsible for the conversion of fructose 1,6-bisphosphate into fructose 6-phosphate (Figure 2). A severe intracellular multiplication defect of a Δ*glpX* mutant was also observed when cells were supplemented with a gluconeogenic substrate (e.g., pyruvate or glycerol). In contrast, wild-type multiplication was restored when the medium was supplemented with glucose. In chemically defined medium (CDM), inactivation of *glpX* also led to a severe growth defect in all the media containing gluconeogenic substrates. Growth of the wild-type strain in CDM lacking glucose (CDMΔGlc) was significantly reduced but still detectable, due to the presence of the amino acids present in the 3 mM range whereas growth of the Δ*glpX* mutant was essentially abolished. Supplementation of CDMΔGlc with individually added excess amino acid (25 mM) improved to variable extents growth of the wild-type strain in the absence of glucose. Remarkably, alanine was the only amino acid to restore wild-type growth (Brissac et al., 2015) although no gene encoding a putative alanine dehydrogenase (catalyzing the conversion of L-alanine to pyruvate) could be predicted in the *F. novicida* genome (unpublished observation). As expected, supplementation of the CDMΔGlc medium with any of the (individually added) twenty amino acids failed to restore growth of the Δ*glpX* mutant, strongly suggesting that amino acids served as gluconeogenic substrates by *Francisella*.

Isotopic profiling, using either ^13^C-labeled glucose or ^13^C-labeled pyruvate, revealed that *Francisella* possessed both active glycolysis and gluconeogenesis pathways in CDM (Brissac et al., 2015). The existence of a complete and functional glycolytic pathway in *Francisella* is in agreement with a genome-wide study which notably revealed that gene *FTN_1210* in *F. novicida* most likely encoded a phosphofructokinase, responsible for the conversion fructose-6P to fructose-1,6P (Enstrom et al., 2012). Indeed, this study, aimed at attributing novel non-predictable metabolic functions to non-essential genes, showed that inactivation of gene *FTN_1210* abolished growth on sugars but not on short-chain carbon sources, suggesting a block in glycolysis. Furthermore, the gene *FTN_1210* was shown to functionally complement the growth defect of an *E. coli pfk* mutant on sorbitol as the carbon source. Of note, the gene *FTN_1210*, now designated *pfk*, is still erroneously annotated as encoding a putative ribokinase in the KEGG database.

Our metabolomics analyses further indicated that the enzyme of the non-oxidative PPP (transketolase, transaldolase) were functional (Figure 2). However, 6-phosphogluconate (6-PG) and pentose phosphates were not detected, suggesting an absence of the oxidative part of PPP, from 6-PG to pentose-5-phospate, suggesting a major role of the Embden–Meyerhof (glycolysis) pathway in glucose catabolism in *Francisella*. Remarkably, a reduction in the intracellular glucose concentration was recorded in both J774.1 and THP-1 macrophages infected for 24 h with *F. tularensis* LVS (Brissac et al., 2015). The need for cytosolic *Francisella* to possess an active gluconeogenic pathway is thus consistent with the significant reduction in the available intracellular glucose pool observed upon infection. The control of the intracellular glucose homeostasis is likely to be a key issue for proper bacterial multiplication. Infected cells might modify their metabolism to reduce the available glucose intracellular pool and, hence, limit bacterial proliferation. Of particular interest, a very recent study (Sanman et al., 2016) demonstrated that *Salmonella typhimium* activated the NLRP3 inflammasome by disrupting the glycolytic flux upon infection of bone marrow-derived macrophages. The authors showed that this trigger occurred because intracellular bacteria were using the macrophage supply of glycolysis precursor molecules. This study suggests that glycolytic disruption may constitute a more general mechanism of inflammasome activation triggered in response to metabolic parasitism by microbes.

**Figure 3:**
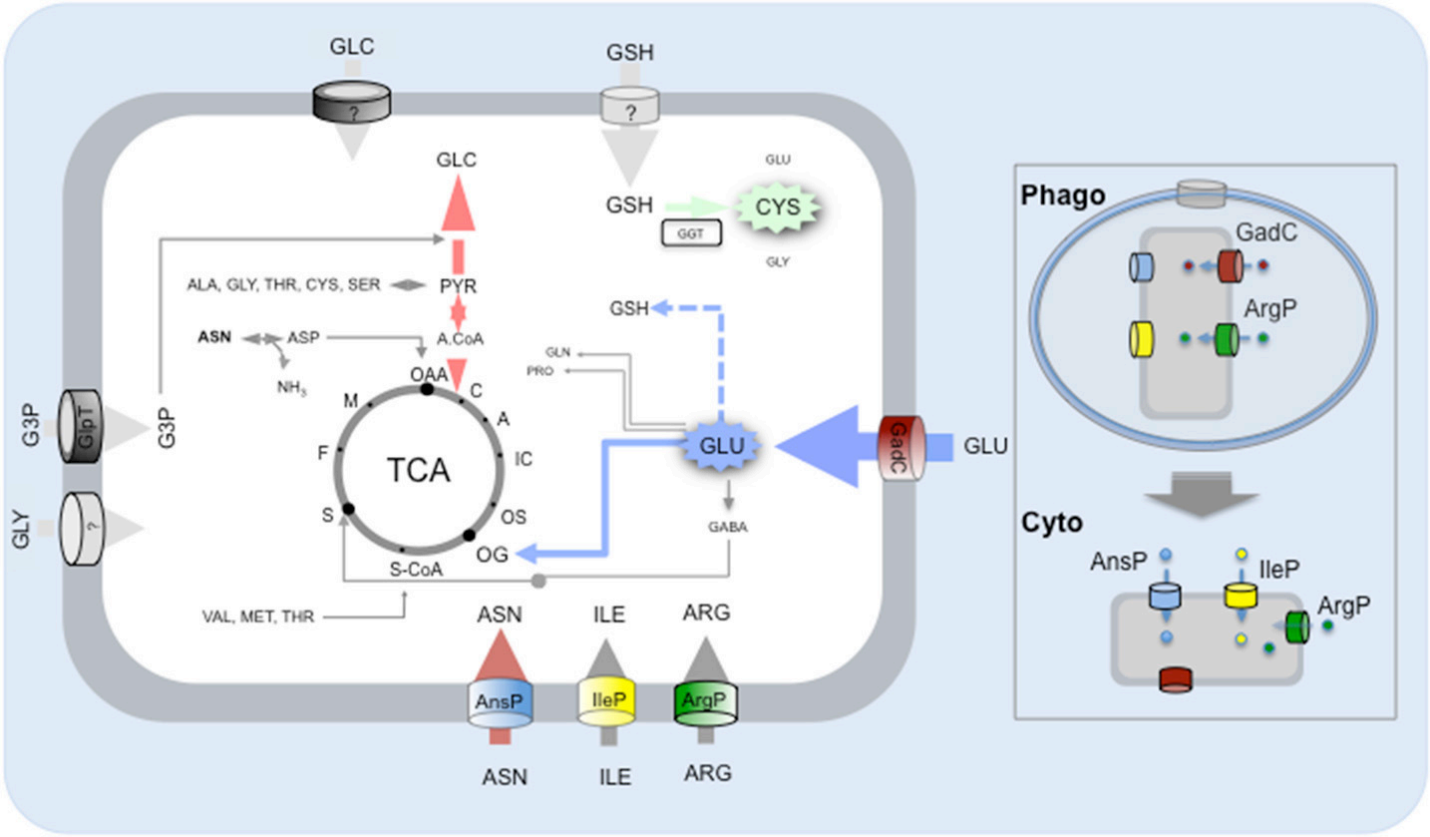
**Amino acid and carbohydrate uptake systems involved in ***Francisella*** intracellular survival**. Schematic depiction of the carbohydrate and amino acid transporters (predicted or characterized) of *Francisella* and contribution of the imported amino acid and carbohydrates to metabolic pathways. Amino acids (ASN, asparagine; ILE, Isoleucine; ARG, Arginine; GLU, Glutamate) are taken up by *Francisella* via dedicated amino acid transporters and used to feed the TCA cycle (GLU) and/or as gluconeogenic substrates and/or as building blocks for protein synthesis (ASN, ARG, ILE). The imported carbohydrates (GLC, Glucose; GLY, Glycerol; G3P, Glyceraldehyde-3-Phosphate) are used to feed the glycolysis/gluconeogenesis pathways. Protein numbers of the represented transporters in *F. novicida*: GadC (FTN_ 0571), AnsP (FTN_1586), IleP (FTN_1654), ArgP (FTN-0848); G3P transporter GlpT (FTN_0636). Boxed to the right of the Figure, the respective contributions of the amino acid transporters to: (i) phagosomal survival and escape (upper part); (ii) cytosolic multiplication (lower part).

In conclusion, our recent studies have shown that amino acid uptake systems and carbohydrate metabolic pathways played both an important and complementary role in *Francisella* pathogenesis (Figure 3). Novel approaches are developed to translate multi-omics data into functional metabolic programs. For example, constraints-based systems analysis has been used on *Francisella* to integrate existing high-throughput data, *in silico* and experimental information (Raghunathan et al., 2010). This approach allowed the reconstruction of a genome-scale metabolic model, which identified significant changes of metabolism during *Francisella* intracellular growth in the infected macrophage. Altogether, a switch from oxidative metabolism, in the initial stages of infection, to glycolysis, fatty acid oxidation, and gluconeogenesis, during the later stages, could be deduced from their analyses.

Understanding the subtle interplay between bacterial and host metabolism will be a major challenge for future studies. Many important questions remain to be answered on the bacterial side to assess the contribution of other nutrient sources (such as lipids, ions) during intracellular multiplication. The molecular dissection of the links between metabolic alterations triggered by the pathogen and the modulation of the innate immune response constitutes a novel and very promising field of investigation.

## Author contributions

AC and JZ wrote the paper, MB edited the paper.

### Conflict of interest statement

The authors declare that the research was conducted in the absence of any commercial or financial relationships that could be construed as a potential conflict of interest.
